# Self-Renewal and Cancers of the Gastric Epithelium: An Update and the Role of the Lectin TFF1 as an Antral Tumor Suppressor

**DOI:** 10.3390/ijms23105377

**Published:** 2022-05-11

**Authors:** Werner Hoffmann

**Affiliations:** Institute of Molecular Biology and Medicinal Chemistry, Otto-von-Guericke University Magdeburg, Leipziger Str. 44, 39120 Magdeburg, Germany; werner.hoffmann@med.ovgu.de

**Keywords:** gastric cancer, gastric self-renewal, gastric mucosa, stem cell, trefoil factor, metaplasia, cell differentiation, lectin, stomach, telocyte

## Abstract

In 2020, gastric cancer was the fourth leading cause of cancer deaths globally. About 90% of gastric cancers are sporadic and the vast majority are correlated with *Helicobacter pylori* infection; whereas familial clustering is observed in about 10% of cases. Gastric cancer is now considered to be a disease originating from dysregulated self-renewal of the gastric glands in the setting of an inflammatory environment. The human stomach contains two types of gastric units, which show bi-directional self-renewal from a complex variety of stem cells. This review focuses on recent progress concerning the characterization of the different stem cell populations and the mainly mesenchymal signals triggering their stepwise differentiation as well as the genesis of pre-cancerous lesions and carcinogenesis. Furthermore, a model is presented (Lectin-triggered Receptor Blocking Hypothesis) explaining the role of the lectin TFF1 as an antral tumor suppressor possibly regulating Lgr5^+^ antral stem cells in a paracrine or maybe autocrine fashion, with neighboring antral gland cells having a role as niche cells.

## 1. Introduction

The stomach is an evolutionarily old complex organ, whose exceptional function is to create an acidic milieu [1]. There are indications that, in the Drosophila stomach-like entity, acid-secreting parietal cells may already exist (review: [2]). The anatomy of the stomach showed great variations during its evolution, with the human glandular stomach consisting of three different anatomical zones, i.e., the cardiac zone, the fundus/corpus zone, and the antral/pyloric zone, whereas the mouse contains, additionally, a non-glandular forestomach. Histologically, these zones contain two different types of gastric glands, i.e., fundic units in the corpus/fundus region and antral units in both the cardiac and the antral/pyloric zones (reviews: [1,3,4]). The luminal surface of the gastric mucosa and its glands is covered by a single-layered columnar epithelium consisting of different cell types, such as surface mucous cells (SMCs), parietal cells, mucous neck cells (MNCs), zymogenic/chief cells, antral gland cells (AGCs), and various classes of endocrine cells (reviews: [1,4,5]). These different epithelial cell types are responsible for, e.g., the secretion of gastric acid (HCl), mucins, and enzymes as well as the endocrine secretion of various hormones.

In order to keep this epithelium functionally active for life, it undergoes continuous self-renewal from stem and precursor cells (reviews: [1,4,6,7]). Similar principles were observed in the Drosophila proventriculus (review: [2]). Gastric self-renewal is regulated by complex reciprocal interactions of the epithelium with underlying mesenchymal cells. The epithelial–mesenchymal interactions already play an essential role during stomach development and many molecular mechanisms are conserved in the adult stomach (reviews: [1,8,9]). Generally, these mechanisms resemble those in the well-studied intestine (review: [10]), the antrum showing more similarities with the intestine than the corpus.

Dysregulation during stomach development can lead to (congenital) gastric disorders (reviews: [1,8]), whereas dysregulated gastric self-renewal in the adult is a known reason for pre-cancerous conditions (metaplasias) and gastric cancer (reviews: [1,11]).

Secretory trefoil factor family (TFF) peptides have lectin activities and diverse molecular functions for mucus barrier protection; their expression is regulated by inflammatory mediators, and they also affect inflammatory processes (reviews: [12,13]). In particular, TFF1 (previously termed pS2) is secreted together with the mucin MUC5AC by gastric SMCs. *Tff1*-deficient (*Tff1*^KO^) mice obligatorily develop adenomas in the gastric antral/pyloric mucosa, and about 30% progress to carcinomas [14]. Thus, *Tff1*^KO^ animals are an established mouse model for spontaneous gastric carcinogenesis and *Tff1* is considered a gastric tumor suppressor gene in the mouse [15,16].

Here, I will discuss recent progress obtained in the field of gastric self-renewal from stem and precursor cells that has developed since 2015 [7] and provide an update concerning the role of Tff1 as an antral tumor suppressor.

## 2. Gastric Self-Renewal from Stem and Precursor Cells

### 2.1. Cellular Architecture of the Gastric Mucosa

**Figure 1:**
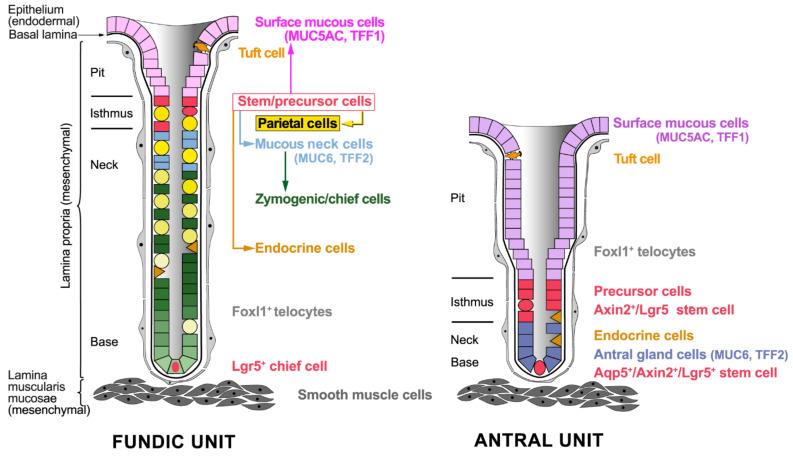
Schematic representation of the gastric mucosa and the two gross types of gastric units in the fundus/corpus and cardia, antrum/pylorus, respectively. A gastric unit consists of the pit (or foveola) and the gland (i.e., isthmus, neck, and base). Shown are the major epithelial (surface mucous, parietal, mucous neck, zymogenic/chief, endocrine, and antral gland cells) and mesenchymal cell types (Foxl1^+^ subepithelial telocytes, smooth muscle cells). The various gastric stem and precursor cells are marked in red. Additionally, figured are rare tuft cells in the pit regions of both units.

The anatomy of the stomach shows variations between human and mouse; the latter is characterized by a non-glandular forestomach (reviews: [1,4,17]). However, the general cellular architecture of the two types of gastric glands in human and mouse is similar, showing subtle differences (Figure 1) [3].

The gastric mucosa and its glands consist of a superficial columnar epithelium (parenchyma), which is of endodermal origin, as well as the stromal cells of the lamina propria and the lamina muscularis mucosae, which are both of mesodermal origin (Figure 1; review: [8]). A basal lamina separates the epithelium from the underlying connective tissue cells of the mesenchyme. Directly underneath the basal lamina there is a network of Foxl1^+^ subepithelial telocytes (Figure 1; previously described as subepithelial myofibroblasts [18] or periglandular fibroblasts [19]), which are thin cells with long protrusions called telopods (review: [20]). Clearly, the expression profile of Foxl1^+^ telocytes differs from that of Foxl1-negative myofibroblasts and they are negative for α-smooth muscle actin [20]. In addition, and characteristic of the alimentary tract, there are several thin layers of smooth muscle cells called the lamina muscularis mucosae, which separates the lamina propria from the submucosa (Figure 1; reviews: [8,18]).

Furthermore, the gastric mucosa is also innervated by the enteric nervous system (not outlined in Figure 1), which is of ectodermal origin, derives from migrating vagal enteric neural crest cells (vENCCs), and populates the gastrointestinal tract via the esophagus (reviews: [1,8]). Signaling triggered by the receptor tyrosine kinase RET and mesenchymal glial-derived neurotrophic factor (GDNF) is critical for this cell migration early in development [1]. Of note is that vENCCs regulate stomach patterning and differentiation [8]. Thus, the proper development and function of the stomach requires complex interactions between cells originating from all three embryonic germ layers.

In addition, the gastric mucosa is capable of showing immune responses. Here, group 2 innate lymphoid cells (ILC2s) play a crucial role as guardians. They make up about 0.47% of viable cells in the murine gastric mucosa, are stimulated by epithelial-derived stress signals such as interleukin (IL)-33, and are the source of Th2 cytokines such as IL-13 [21]. Of special note is that ILC2s are regulated in a sex-specific manner [22].

From the elegant pioneering work of Charles Leblond and his coworkers mainly in the 1980s, it is well known that the murine, and also the human, gastric epithelium continuously renews in a bi-directional way from a highly proliferative region in the isthmus, where stem and precursor cells are located [6,23,24]. Of special note is that antral SMCs show a higher turnover rate than fundic SMCs [6,25] and also the number of proliferating cells is much higher in the antral units when compared with fundic units [26].

Generally, SMCs originate from progenitor cells by differentiation and migration to the gastric pit, whereas parietal cells, MNCs, AGCs, and endocrine cells originate by differentiation and downward migration towards the gastric base. Chief cells originate by transdifferentiation of MNCs and downward migration (reviews: [4,5,7,27]). Notably, the expression profile of SMCs in the fundic and the antral units differs significantly, particularly concerning characteristic secretory proteins [26,27]. Parietal cells are the major organizing centers of fundic units due to their secretion of several epidermal growth factor (EGF) receptor ligands and the morphogen/morphostat Sonic hedgehog (SHH). The latter is characteristic of fundic units and enables a specific interaction with underlying mesenchymal cells (see Section 2.3; reviews: [5,27,28,29,30,31,32]).

### 2.2. Stem Cells of the Gastric Epithelium

In the “classical” phase of stem cell research, in vivo pulse-chase labeling experiments with ^3^H-thymidine followed by radioautography as well as electron microscopy were used to follow up the turnover of the gastric epithelium and to characterize stem and precursor cells by their morphology, i.e., by their undifferentiated appearance lacking a secretory machinery [6,24]. Later on, using genetic and molecular tools allowing lineage tracing, it became increasingly clear that the situation is more complex. Both fundic and antral units differ in their sets of stem cells, with each unit containing more than one stem cell population (reviews: [4,7,17,33]). Epithelial stem cell proliferation is tightly regulated by cells of their microenvironment, the “niche” [4]. Here, reciprocal epithelial–mesenchymal interactions regulate their maintenance (see Section 2.3).

#### 2.2.1. Stem Cells in the Fundic Units

Only within the last years have two different types of stem cells been defined in more detail in the murine fundic units (Figure 1). The pool of actively proliferating cells in the isthmus contains stem cells, which are capable of long-term self-renewal [34]. Here, parietal cells act as physical barriers against the lateral expansion of the isthmus clones, which forces rapid vertical expansion parallel to the gland axis [34]. Generally, all the markers previously claimed to be specific for the isthmus stem cells (such as Sox2, Runx1, Lrig1, Mist1, and Bmi1) showed a very broad pattern of expression [34]. Instead, two marker candidates were identified for this actively cycling isthmus progenitor population, i.e., Mki67 and Stathmin1 (Stmn1) [34]. Recently, Iqgap3 was established as a marker for isthmus stem cells, which also showed strong enrichment for Mki67 and Stmn1, but only low Mist1 expression [35]. However, Mist1^+^ fundic isthmus stem cells were also described as tracing entire fundic units [36]. Generally, the actively proliferating Iqgap3^+^/Stmn1^+^ stem cell population seems to be congruent with that described originally in the isthmus (review: [6]). Of note is that an expansion of isthmus stem cell clones was observed after injury of parietal cells [34], after tamoxifen treatment, or after inflammation [35,36].

Additionally, there are slow-cycling stem cells in the base of fundic units. Here, Lgr5^+^ chief cells (Figure 1) can function as stem cells following injury in mouse and human [37]. This population is probably congruent with previously described Troy^+^ chief cells acting as reserve stem cells [38]. Damage (e.g., by *Helicobacter pylori*) can activate these normally quiescent reserve stem cells, which then act as a key origin for cancer in the corpus after an oncogenic mutation [37].

#### 2.2.2. Stem Cells in the Antral Units

Different types of stem cells were also characterized in the antral units (Figure 1). Originally, Lgr5^+^ stem cells were identified at the base of antral units [39], which were later shown to also be positive for Axin2 (Axin2^+^/Lgr5^+^; [40]) and Aquaporin 5 (Aqp5^+^; [41]). Of note is that these antral Lgr5^+^ stem cells at the gland base divide symmetrically [42].

Additionally, a second type of highly proliferative stem cell (pronounced co-staining with Ki67) was described directly above in the lower isthmus, characterized as being Axin2^+^/Lgr5^−^ [40]. This stem cell population seems to be congruent with that described originally in the isthmus (review: [6]) and might be related to Sox2^+^ [43], Cck2r^+^ [44], and Bmi1^+^ [45] stem cells, respectively, described previously.

Both Axin2^+^/Lgr5^+^ and Axin2^+^/Lgr5^−^ stem cells are capable of repopulating the entire antral gland, but they respond differently to R-spondin 3 [40]. While Axin2^+^/Lgr5^−^ stem cells respond by proliferation [40], Axin2^+^/Lgr5^+^ stem cells respond by differentiation [46] (see Section 2.3). The highly proliferative Axin2^+^/Lgr5^−^ stem cells provide their progeny bi-directionally and can even give rise to Axin2^+^/Lgr5^+^ stem cells at the gland base [40].

### 2.3. Gastric Stem Cell Niches, Reciprocal Epithelial–Mesenchymal Interactions

**Figure 2:**
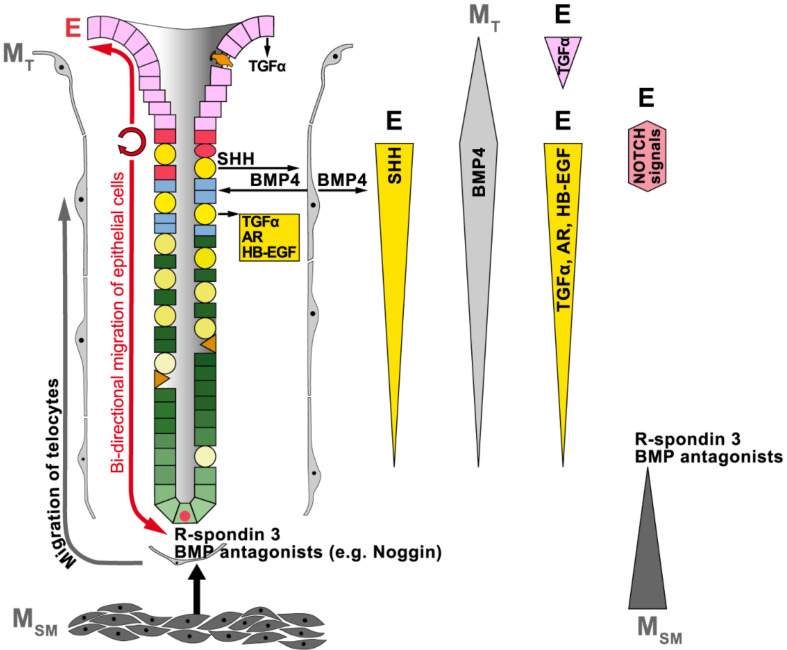
Schematic representation of reciprocal epithelial–mesenchymal interactions in the human gastric fundic unit. Shown are also gradients originating from the epithelium (E) or the mesenchyme (M; telocytes, M_T_; smooth muscle cells, M_SM_) concerning SHH, BMP4, TGFα, AR, and HB-EGF along the gland axis. Furthermore, the mesenchymal expression of R-spondin 3 and BMP antagonists (e.g., Noggin in human) at the gland base, as well as the spatial organization of epithelial NOTCH signaling, is depicted.

Extensive studies of the intestine revealed that the underlying mesenchymal cells not only form the niches for the epithelial stem cells, but also provide essential signals for their differentiation along the gland axis [18,20,47,48,49]. Major players are a rare population of subepithelial Foxl1^+^ telocytes (Figure 1), which form a plexus from the stomach to the colon [20]. Of special note is that the expression pattern of these Foxl1^+^ telocytes probably differs along the gland axis, enabling either proliferation (by expression of WNT activators/ligands) or differentiation (e.g., by expression of various WNT inhibitors) [20,50,51]. Furthermore, these subepithelial telocytes also undergo self-renewal—they migrate and show apoptosis but with a much lower turnover than epithelial cells (Figure 2) [20,52]. For example, in intestinal stem cell niches, the expression of the bone morphogenetic protein (BMP) antagonist Gremlin 1 was demonstrated; here, these cells act as stem cells and expand to renew the mesenchymal sheet [53]. However, gastric and intestinal telocytes/mesenchymal myofibroblasts differ in their expression profiles; gastric mesenchymal myofibroblasts induce differentiation of gastric epithelial cells and are characterized by the expression of growth arrest-specific gene 1 (Gas1) [54], which is a co-receptor for SHH, Indian hedgehog (IHH), and RET [55].

Within the last years, major progress has been made in understanding the complex reciprocal epithelial–mesenchymal interactions regulating gastric epithelial self-renewal. A network of gradients of several morphogens/morphostats triggers complex signaling cascades, which determine the fate of the cells along the gland axis, i.e., proliferation versus differentiation, migration, and apoptosis (Figure 2). Major players are signaling pathways triggered by WNT, NOTCH, hedgehog (HH), transforming growth factor β (TGFβ) superfamily/BMP, and EGF receptor ligands (review: [56]). Many principles are analogous to those established already in the intestine (reviews: [10,18,57,58]).

Key drivers for the proliferation program in stem cells are members of the secreted WNT signaling proteins, which are lipid modified. They act as short-range cellular signals after their release from the stromal stem cell microenvironment, the “niche” [33]. Wnt ligands are of mesenchymal origin, they bind to the receptor complex Frizzled/Lrp5/6 at the stem cells, and their activation is controlled by the Lgr5/R-spondin/Rnf43 module [33] in a similar fashion as previously established for the intestine [58]. There is a variety of Wnt ligands, which can specifically induce signaling via canonical (classical: β-catenin translocation) and non-canonical pathways, respectively.

**Figure 3:**
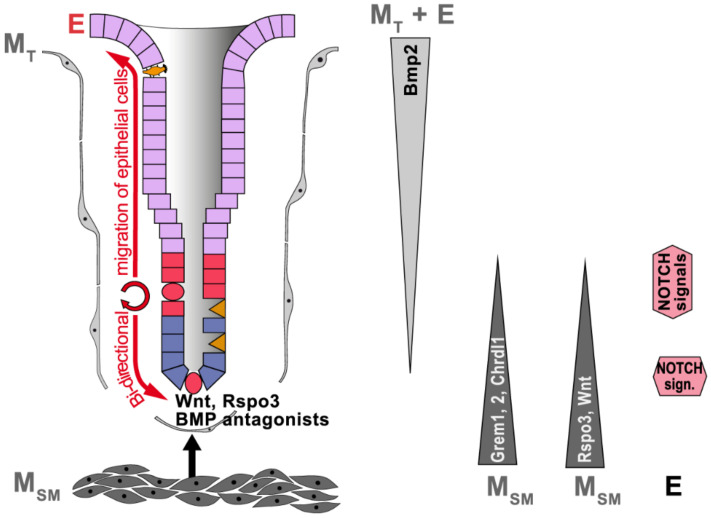
Schematic representation of reciprocal epithelial–mesenchymal interactions in the gastric antral unit as determined for the mouse. Shown are also gradients originating from the epithelium (E) or the mesenchyme (M; telocytes, M_T_; smooth muscle cells, M_SM_) concerning R-spondin 3, Bmp2, the BMP antagonists Gremlin 1, Gremlin 2, and Chordin-like 1 (Chrdl1) along the gland axis. Furthermore, the spatial organization of Notch signaling in the isthmus region and the gland base is depicted.

Furthermore, NOTCH signaling has long been known as another prerequisite for stem cell maintenance, and this has also been demonstrated for gastric stem cells [59,60]. In adult gastric glands, NOTCH signaling is restricted mainly to the proliferative zones in the isthmus of both fundic (Figure 2) and antral glands [59] as well as the base of antral glands (Figure 3) [59,60,61]. NOTCH ligands are transmembrane proteins of epithelial origin allowing juxtacrine interactions with NOTCH receptors; the latter are at stem and precursor cells. The activation of NOTCH 1 and NOTCH 2 receptors promotes stem cell proliferation in both fundic and antral units, whereas their inhibition generally increases cellular differentiation in the antrum, even of SMCs, but not in the corpus (review: [62]). Thus, differentiation of SMCs seems to be regulated differently in the corpus and antrum, respectively. This is in agreement with the observation that SMCs from the human corpus and antrum, respectively, differ characteristically in their expression profiles [26,27].

In the murine corpus, reports of Wnt signaling are scarce and Wnt inhibition did not inhibit corpus proliferation [63]. For example, a source of Wnt is expected in mesenchymal cells and maybe also in Troy^+^ parietal cells near the gland base [38]. Furthermore, stem cells in the murine gastric corpus isthmus were claimed to be supported by a niche of ILC2s secreting the non-canonical ligand Wnt5a [63]. However, it is more reasonable that gastric Wnt5a originates from subepithelial telocytes instead. For example, it is well known that intestinal Foxl1^+^ telocytes synthesize Wnt5a [50]. In contrast, R-spondin 3 (Rspo3), which is a critical regulator of the Lgr5 signature genes, is clearly expressed by smooth muscle cells of the muscularis mucosae just beneath the gland base (Figure 2) [46]. This points to these mesenchymal cells being the major determinants for Lgr5^+^ reserve stem cells at the gland base.

In contrast, the two stem cell populations in the antrum are controlled by Wnt and R-spondin signaling but in different ways [33]. In the murine antrum, Wnt3a, Wnt4, and Wnt5a expression is located in the isthmus and midglandular compartment, whereas strong Wnt11 expression was detectable throughout the gland [40]. Furthermore, soluble Wnt inhibitors (FRP1, DKK1, DKK3) are expressed in the lamina propria between the glands (probably Foxl1^−^ telocytes) as shown in human mucosoid cultures in vitro [64]. Generally, telocyte markers, such as Wnt ligands and the HH downstream transcription factor Gli2, are conserved between stomach and intestine [65]. Furthermore, by analogy to the intestine [58], Rspo3 is produced by smooth muscle cells in the muscularis mucosae directly beneath the gland base (Figure 3) [40]. Surprisingly, only Axin2^+^/Lgr5^−^ stem cells in the isthmus respond to Rspo3 by proliferation [40]. In addition, Rspo3 is essential for the regeneration of Lgr5^+^ cells from Axin2^+^/Lgr5^−^ stem cells [46]. In contrast, Rspo3 failed to induce proliferation in Axin2^+^/Lgr5^+^ stem cells at the gland base in spite of them being exposed to the highest Rspo3 concentration [46]. Basal Lgr5^+^ cells, rather, showed differentiation towards secretory cell types, with five clusters identified [46]. Notably, cluster 2 was characterized by expression of the peptide Tff1 [46].

SHH and BMPs are extensively studied signaling systems mediating complex reciprocal crosstalk between the epithelium and the mesenchyme in fundic units (Figure 2; reviews: [3,5,27]). Of note is that SHH/BMP4 are negative regulators of gland cell, but not pit cell proliferation. SHH is a basolateral secretory product of parietal cells, whose complex biosynthesis and processing is dependent on the acidic environment on the apical side; SHH predominantly activates underlying mesenchymal telocytes, but also epithelial cells, via receptors (reviews: [30,31,66]). BMP4 is one of the SHH target genes and is mainly released from activated mesenchymal cells of the isthmus (Figure 2) [67], where it acts via receptors (BMPR1 and BMPR2) and in turn plays a major role for differentiation of epithelial cells along the fundic gland axis, i.e., parietal, mucous neck and chief cells (review: [68]). Of special note is that BMPs can also activate signaling cascades within mesenchymal cells as BMPR1 is also located on mesenchymal cells [69]. The action of BMPs is blocked by specific inhibitors, such as Noggin, Gremlin, and Chordin [68]. For example, Noggin is expressed by smooth muscle cells in the lamina muscularis mucosae in proximity to the base of human gastric fundic glands (Figure 2) [67]. Taken together, SHH/BMP4 regulate differentiation processes along the fundic gland axis. Of special note is that SHH expression is inhibited by IL-1β, explaining why inflammation dysregulates fundic self-renewal (review: [70]).

The situation in antral units is somewhat different and has been particularly investigated in the murine stomach [71]. Here, the BMP antagonists Gremlin 1, Gremlin 2, and Chordin-like 1 were expressed in the lamina muscularis mucosae beneath the gland base, whereas the predominant BMP Bmp2 was expressed in Foxl1^+^ telocytes and probably also epithelial cells, predominantly at the gland surface (Figure 3) [71]. In contrast, Bmp4 transcripts were mainly detected in the middle of the glands [71]. Generally, BMP signaling promotes the differentiation of stem cells towards SMCs. This process is even up-regulated by a positive auto- and paracrine BMP positive feedback loop [71]. Bmp2 also decreased Rspo3 expression [71], which clearly spatially organizes cell differentiation towards SMCs in the pits. Thus, predominant Bmp2 signaling in the surface of antral glands creates an organizing center restricting cell proliferation to the isthmus and the gland base. A similar principle was already described for the intestine [47].

EGF receptor ligands such as transforming growth factor α (TGFα), amphiregulin (AR), and heparin-binding EGF (HB-EGF) are important proliferation and differentiation signals, each having a specific physiological response (Figure 2; reviews: [5,27]). These three ligands are basolaterally secreted from parietal cells in fundic units, whereas TGFα is additionally released from superficial SMCs (reviews: [27,72]).

By analogy with the intestine [10,57], further gradients are probably necessary for proper differentiation of gastric glands, e.g., Eph receptors and ephrins [73] as well as the phosphatase PTEN. Another interesting player is Indian hedgehog (IHH), which is expressed in SMCs with an increasing gradient from the fundus to the antrum [66] and mediates gastrin-induced epithelial proliferation [74].

Knowledge of the different gradients and studying the molecular mechanisms triggered is a prerequisite to understanding how the different types of glands in the fundus and antrum are generated from stem cells and how their continuous self-renewal is regulated. This knowledge was successfully applied to generate fundic and antral 3D organoids, respectively, e.g., from the directed differentiation of human pluripotent stem cells (hPSCs) or murine somatic stem cells (mSSCs) (reviews: [75,76,77]. Furthermore, even the differentiation of certain epithelial cell types is now possible in vitro (mucosoid cultures) [64,67].

These new technologies are used now to investigate pre-cancerous conditions [67], to establish models of infectious diseases, such as infection with *H. pylori* [64,78], and to transplant even specific cancer organoids in mouse models to generate tumors with accompanying metastases and test different chemotherapy regimens [79].

## 3. Gastric Cancers and Their Pre-Cancerous Lesions

In 2020, gastric cancer (GC) was the fifth most frequently diagnosed cancer globally and the fourth leading cause of cancer deaths [80]. Historically, gastric carcinomas were divided by their histological phenotype into a minor (“heterogeneous”) and two major types, i.e., the “intestinal-type” and the “diffuse” (Laurén classification, 1965), the intestinal-type representing the predominant form (>50%) (review: [81]). In 1988, a consecutive sequence of pre-cancerous conditions was reported as leading to intestinal-type cancer (known as “Correa cascade”; review: [82]). Only in 1992 was this model updated to include inflammation (chronic atrophic gastritis) as a prerequisite for carcinogenesis and *H. pylori* as the major source of inflammation (reviews: [70,82,83]).

In 2010, five major types of gastric carcinomas were recognized by the World Health Organization (WHO), i.e., papillary, tubular and mucinous adenocarcinoma, poorly cohesive carcinoma (including signet-ring carcinomas), and mixed carcinomas. Most GCs are sporadic (about 90%), and familial clustering is observed in about 10% of the cases [84]. Only about 1–3% of cases are really hereditary (Carneiro 2012). Most hereditary diffuse GCs are caused by mutations in the E-cadherin gene (CDH1). Taken together, GC is a result of environmental effects and genetic predispositions [85]. As a step towards precision medicine, a molecular characterization of gastric adenocarcinomas was proposed by The Cancer Genome Atlas (TCGA) research network in 2014 [86].

### 3.1. Helicobacter pylori and Its Colonization of Gastric Glands

About 75–90% of sporadic GCs are correlated with *H. pylori* infection, whereas Epstein-Barr virus accounts for about 5–10% (review: [70]). Generally, there are three main gastric infection phenotypes known with different clinical outcomes (review: [87,88]). The most common phenotype is characterized by a mild pan-gastritis with no serious disease. The second phenotype (about 15% of cases) shows an antrum-predominant pattern of *H. pylori* infection and gastritis resulting in hyperchlorhydria and the development of peptic ulcers, particularly duodenal and pre-pyloric gastric ulcers. In contrast, in the third and most serious phenotype (1% of subjects), a corpus-predominant infection leads to hypochlorhydria due to a loss of parietal cells, corpus-predominant inflammation, mucosal atrophy (atrophic gastritis), intestinal metaplasia (IM), and, eventually, intestinal-type adenocarcinomas along the Correa pathway (reviews: [83,87,88]). IM is defined by a replacement of gastric cells by intestinal cells, such as goblet cells (review: [89]).

For a long time, *H. pylori* was described as being restricted to the mucus layer covering the SMCs in the pits as well as adhering to these cells [88]. Only later on has it been demonstrated that *H. pylori* is also able to invade gastric glands and to directly interact with gastric stem and progenitor cells in both human fundic and antral units [90]. In a murine model, *H. pylori* infection stimulated the increased secretion of Rspo3 not only from smooth muscle cells underneath the antral gland base (Figure 3), but also from telocytes between the antral glands [40], both of which induced expansion of the Axin2^+^ cell pool, in particular by proliferation of the Axin2^+^/Lgr^−^ stem cells in the isthmus [40]. This resulted in accelerated turnover of the antral glands and also an increased number of Axin2^+^/Lgr5^+^ stem cells leading to gastric gland hyperplasia [90]. In contrast, in the basal Lgr5^+^ cells, Rspo3 induced differentiation into antimicrobial cells, which secrete intelectin-1, limiting the growth of *H. pylori* [46]. Thus, Rspo3 is also crucial for defense against *H. pylori* in the gland base to counterbalance gland colonization [46].

Furthermore, *H. pylori* infection strongly down-regulated Bmp2 expression in both mesenchymal and epithelial cells at the antral gland surface, whereas expression of the BMP inhibitors Gremlin 2 and Chordin-like 1 was up-regulated in mesenchymal cells beneath the gland base as well as in telocytes (Figure 3) [71]. This effect was particularly strong in the proximal antrum and the transitional zone between the corpus and antrum, which is preferentially colonized by *H. pylori* and shows prominent morphological changes [71]. In gastric organoids, the Bmp2 down-regulation was shown to be triggered by interferon-γ (IFN-γ), which is released by infiltrating T cells after *H. pylori* infection [71]. Moreover, an up-regulation of both Rspo3 and Lgr5 was observed in IFN-γ-treated gastric organoids [71]. Taken together, after *H. pylori* infection and the immune response by T cells, the release of IFN-γ is essential to trigger morphological changes in antral units towards the proliferation of stem cells and expansion of mucous gland cells, the latter expressing Muc6 and Tff2 (Figure 1). Of special note is that the development of hyperplasia as well as IFN-γ signaling depend on a functional type 4 secretion system (T4SS) of *H. pylori* [71,90], i.e., translocation of the virulence factor/oncoprotein CagA (review: [91]) is essential.

Remarkably, the intrinsic combination of MUC6 and TFF2, both secreted from MNCs, AGCs (Figure 1), and duodenal Brunner gland cells (details: [12,92]), seems to protect nearby gastric stem cell populations from infection with *H. pylori*. The mucin MUC6 contains an *N*-acetylglucosamine (GlcNAc) residue at the non-reducing terminal of its O-linked glycan, which is recognized by the monoclonal antibody HIK1083 as well as the lectin GSA-II from *Griffonia* *simplicifolia* (review: [93]). This unusual sugar residue, which is conserved in MUC6 from frog to human, plays an important role in the gastric/duodenal mucosal innate immune defense by inhibiting cholesterol α-glucosyltransferase from *H. pylori* and thereby suppressing *H. pylori* growth [94]. This enzyme allows depletion of cholesterol from the plasma membrane of the host cells, leading to the disruption of lipid rafts and diminished IFN-γ signaling, allowing *H. pylori* to persist despite ongoing inflammation [94]. The key enzyme for the synthesis of the terminal αGlcNAc is α1,4-*N*-acetylglucosaminyltransferase (A4GNT); *A4gnt^KO^* mice obligatorily develop antral adenocarcinoma along a hyperplasia–dysplasia–carcinoma sequence with an inflammatory phenotype [95]. This implies a role for αGlcNAc as an antral tumor suppressor (see also Section 4.5).

TFF2 is a MUC6-binding secretory lectin characteristic of MNCs and AGCs (see Figure 1) and plays a role in the mucosal innate immune defense by physically stabilizing the MUC6 matrix in the inner mucus layer [12,92,96,97]. This explains why Tff2-deficient mice show accelerated progression of *H. pylori*-induced gastritis to dysplasia [98].

### 3.2. Fundic Intestinal-Type Adenocarcinomas: Atrophic Gastritis, Metaplasias, Inflammation

As IM develops in the setting of atrophic gastritis and obviously plays a major role as an intermediate step in the development of gastric cancer (pre-neoplasia, pre-cancerous condition) of the intestinal-type [82,83], it was a major aim to understand the genesis of IM. Only within the last two decades has it become increasingly clear how IM develops in the fundic units in the settings of inflammation (review: [11]). This multistep process was investigated mainly using murine models [99].

A hallmark was the discovery of another metaplasia in 1999, which replaces zymogenic chief cells at the base of fundic glands by Spasmolytic Polypeptide (i.e., TFF2) expressing metaplastic (SPEM) cells [100]. Metaplastic glands with SPEM cells at the gland base are termed “pyloric metaplastic glands” [11]. Generally, SPEM is strongly associated with gastric adenocarcinoma [100]. SPEM cells contain both mucous and zymogen secretory granules, but they can be clearly distinguished from mucous and zymogenic/chief cells by the expression of WFDC2, which is a secretory protein also known as human epididymis protein 4 (HE4) [11,101]. In short, SPEM cells seem to derive from plastic chief cells at the gland base being re-programmed to a mixed phenotype with mucous neck cells, where expression of TFF2 and MUC6 is up-regulated again [11]. Alternatively, a dysregulated transdifferentiation of MNCs/pre-zymogenic cells is discussed [27]. The latter would be in agreement with the report that SPEM cells can arise from isthmal progenitors and MNCs [102,103]. However, Lgr5^+^ chief cells (see Figure 1) were excluded as the origin of SPEM cells [104]. The next step is the re-entry of such post-mitotic SPEM cells into the cell cycle by re-activating mTORC1; this process towards proliferation was termed “paligenosis” [105]. Of special note is that the induction of SPEM is accompanied by the accumulation of intracellular double-stranded RNA (dsRNA) and the up-regulated expression of the adenosine RNA deaminase ADAR1, which gives license to the cells to proliferate (pro-survival role) [106].

The next consecutive step is the timely delayed progression from SPEM to IM under the settings of chronic inflammation. This has been documented in different mouse models [11]. In addition, a model using a *Claudin18-IRES-CreERT2* driver to introduce conditional mutations specifically in the gastric epithelium resulted in early time points in the generation of SPEM with transition to IM as disease progressed to metastatic, chromosomal-instable-type gastric cancer [79].

A major question arises as to how the generation of SPEM cells is triggered. Generally, SPEM cells arise in the context of both chronic inflammation and parietal cell loss. Typical experimental inducers of SPEM in murine model systems are *H. pylori* infection and parietal cell-toxic drugs (protonophores such as DMP-777, which also inhibits inflammation, and its analogue L-635, which does not inhibit inflammation [99,107]). Furthermore, very different transgenic mouse models spontaneously develop SPEM, such as amphiregulin-deficient mice, which also develop IM [108], and *Bmpr1a*^ΔMES^-deficient mice [69]. These are indications that proper functioning of parietal cells (e.g., secretion of H^+^ and amphiregulin) is a prerequisite for their role as organizing centers of fundic units and is essential for preventing SPEM; such a role also includes complex mesenchymal–mesenchymal interactions via BMP4, which are obviously essential for the maintenance of proper epithelial self-renewal.

By comparing the effects of DMP-777 and L-635 in murine models, it became clear that a type 2 inflammation plays a major role in the progression of SPEM to proliferative metaplasia [109]. In a series of systematic studies, James Goldenring and his coworkers showed that the metaplastic process depends on a signaling cascade, where the infiltration of M2a macrophages, the release of the alarmin IL-33, the activation of already present gastric ILC2s by IL-33, and the release of the Th2 cytokine IL-13 by ILC2s are involved (review: [11]). Notably, after a lesion, the release of IL-33 can probably be triggered by extracellular TFF2 (details: [13]). In an autoimmune gastritis model, mast cells were identified as the predominant sources of IL-13 [103]. Ablation of ILC2s or deletion of IL-33 or IL-13 resulted in significantly fewer SPEM cells after parietal cell loss [21,107]. Furthermore, after ablation of ILC2s, the expansion of SMCs (foveolar hyperplasia) and tuft cells is attenuated; the latter express IL-25, which can also stimulate ILC2s [21]. Currently, a direct activation of multiple gastric epithelial cells (including chief cells) by IL-13 is likely to promote metaplasia development [103,107].

The inhibition of gastric acid secretion by drugs is also clinically important. The pharmacologically relevant proton pump inhibitor (PPI) omeprazole resulted in a significant reduction in SHH expression [110]. Loss of SHH expression in parietal cells leads to SPEM [111]. This implies that there is a link between acid suppression and metaplastic changes. This view is supported by the observation that omeprazole changed the dynamic features of parietal cells in a rabbit model [112]. Long-term use of PPI has been reported to be a risk factor for gastric cancer [113].

In an experimental murine model, it has been demonstrated that a *Kras^G12D^* mutation in Mist1^+^ fundic isthmus stem cells gives rise to metaplastic foci in the isthmus, which moved down to the gland base and replaced the gland with IM and dysplasia [63]. Of special note is that the constitutive activation of NOTCH resulted in the activation of intestinal-type GC, whereas the activation of WNT signaling did not show this effect [63]. Taken together, NOTCH signaling, but not WNT signaling, is an oncogenic pathway for fundic Mist1^+^ isthmus stem cells towards intestinal-type gastric cancer [63].

However, fundic Lgr5^+^ chief cells (reserve stem cells) can also be the origin of gastric cancer after a *Kras^G12D^* mutation [37]. These fundic Lgr5^+^ chief cells, located along the lesser curvature, are also able to be the origin of SPEM, but only after inflammation (infection with *H. felis*) plus inhibition of BMP signaling [114]. This is in contrast to the previous model, where SPEM was induced by DMP-777 or L-635 [104].

### 3.3. Diffuse Gastric Adenocarcinomas

GC of the diffuse type is rather rare and characterized by pan-gastritis throughout the stomach but no atrophy [83]. CDH1 mutations are frequently found in this type, which is restricted to the gastric fundus/corpus [84]. In an experimental mouse model, it was demonstrated that a loss of *Cdh1* in Mist1^+^ fundic isthmus stem cells (*Cdh1^ΔMist1^*) resulted temporally in the formation of atypical cells with signet-ring morphology [63]. In the setting of chronic inflammation (*H. felis* infection), atypical foci were preserved; only the addition of a *Trp53* mutation led to invasive diffuse GC [63]. The administration of anti-inflammatory dexamethasone reduced the atypical foci to the control level [63]. This indicates that the development of diffuse GC from fundic Mist1^+^ stem cells is dependent on chronic inflammation and recapitulates the pathogenesis of a signet-ring carcinoma [63].

Recently, gastric carcinomas of the diffuse type were described, which may originate from enterochromaffin-like (ECL) cells [115].

### 3.4. Neoplasms of the Gastric Antrum

A rare class of neoplasm is the pyloric gland adenoma, which is often seen in the settings of familial adenomatous polyposis, etc. [116]. Furthermore, a number of cases of *H. pylori*-negative differentiated adenocarcinoma located in the antrum were described [117].

### 3.5. Mechanisms of Field Cancerization: Monoclonal Conversion, Gland Fission

As discussed in Section 3.2 and Section 3.3, metaplasia and carcinoma originate from individual cells and then expand within the entire gland affected. Thus, the question arises how they expand within the stomach. The clonal origin of human gastric units was confirmed by the visualization of mutations in the cytochrome c oxidase of single units [118]. The spreading of a mutation all over the entire gland is called monoclonal conversion [118]. Furthermore, patches of entirely mutated gastric units were observed, which developed by budding from the isthmus/neck region, i.e., by gland fission [118]. Analysis of IM in the human stomach also revealed patches that developed through gland fission [119]. Furthermore, when specific mutations, such as APC or TP53, occur in an intestinal metaplastic gland, it becomes dysplastic and expands (dysplastic field) and the lesion becomes genetically diverse over time, indicating an evolution towards a carcinoma [119].

## 4. TFF1 Is an Antral Tumor Suppressor

### 4.1. The Secretory Lectin Trefoil Factor Family 1 (TFF1)

**Figure 4:**
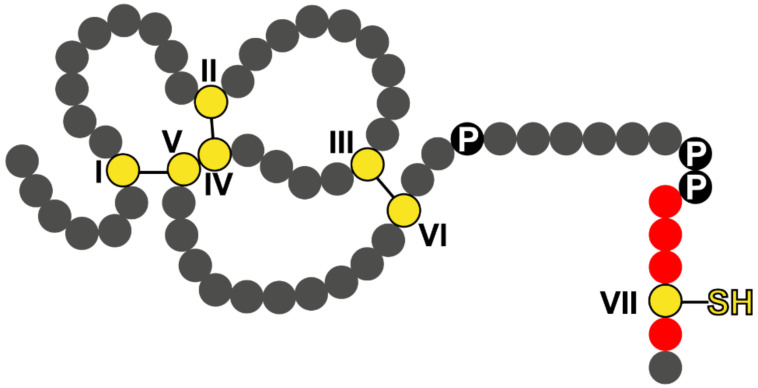
Schematic structure of human TFF1. Cysteine residues (numbering in Roman numerals) are shown in yellow. Cys^I^^–V^, Cys^II^^–IV^, and Cys^III^^–VI^ within the TFF domain are linked by three disulfide bridges, whereas Cys^VII^ outside the TFF domain contains a free thiol group. Acidic residues in the proximity to Cys^VII^ are shown in red. Additionally outlined are the proline residues (P) at the C-terminal outside the TFF domain.

TFF1 is a remarkable secretory peptide typical of gastric SMCs, which contains seven cysteine residues [120,121]. Surprisingly, a major proportion of gastric TFF1 exists as a monomer, as shown for human [122], mouse [123], and *Xenopus laevis* (ortholog xP1) [124]. Minor forms are disulfide-linked hetero-dimers with gastrokine 2 (Gkn2), IgG Fc binding protein (FCGBP), and an unknown protein X (M_r_: 60k), respectively, as well as a homodimer [122,123]. Cys^I^ to Cys^VI^ form three characteristic intramolecular disulfide bridges, which are typical of the TFF domain (Figure 4; reviews: [121,125,126], whereas Cys^VII^ has a highly exposed free thiol group (Figure 4). This is unusual, as secretory peptides are normally devoid of free thiol groups as they are the subject of assembly, retention, or degradation in the endoplasmic reticulum (ER) [127]. In analogy to Ig light chains, the four acid residues in direct proximity to Cys^VII^ probably enable TFF1 to escape assembly, retention, or degradation in the ER; in addition, high nucleophilicity of Cys^VII^ is expected (details: [12,122,123,124]). This leads to the hypothesis that TFF1 could serve as an extracellular scavenger for reactive oxygen/nitrogen species (ROS/RNS), which would be particularly important in the acid environment of the stomach where H_2_O_2_ and nitric oxide (NO) are formed (details: [12,122,123,124]).

Of special note is that synthetic homodimeric TFF1 is able to bind in vitro to gastric mucins from human, pig, and *X. laevis*, probably as a lectin [128]. In human, the GSA-II positive mucin MUC6, but not MUC5AC, was clearly identified as the target [122]. There are indications that the sugar epitope recognized in MUC6 is similar, but not identical, to that recognized by TFF2; a GlcNAc residue being an essential part of the epitope recognized by TFF1 (details: [12,126]).

In addition, dimeric TFF1 also binds as a lectin to the core oligosaccharide of wild type *H. pylori* [129], but not to the mutant strain P12ΔHP0857 lacking sedoheptulose 7-phosphate isomerase [128]. This points again to GlcNAc as being part of the sugar epitope recognized by TFF1.

At relatively high concentrations (10^−5^ M), TFF1 lowers cell proliferation by delaying G1-S phase transition and reduces apoptosis [130]. Furthermore, TFF1 is differentially expressed in stationary and migratory rat gastric epithelial cells, and Tff1-siRNA negatively influences migration of these cells [131]; this points to a weak motogenic effect of TFF1, e.g., enhancing gastric restitution after damage. Combined motogenic and anti-apoptotic effects synergistically support the important process of cell migration, i.e., such a connection makes sure that migrating cells do not die. This explains why both mechanisms are coordinately regulated [132].

Furthermore, TFF1 is down-regulated during gastric inflammation, whereas it is ectopically expressed in various other chronic inflammatory diseases (review: [13]). For example, TFF1 synthesis is induced in mucosal ulcerations during Crohn’s disease [133], and cerebral TFF1 expression is increased in two murine models of neuroinflammation [134].

### 4.2. Results from Tff1^KO^ Mice

Generally, *Tff1^KO^* mice show a severe phenotype [14,15]. In all *Tff1^KO^* mice, the antral/pyloric mucosa was thicker already at 3 weeks of age [14]. At 5 months of age, the antral mucosa showed a 10-fold increase in the mitotic index with hypertrophic elongated pits, severe hyperplasia, and high-grade dysplasia along the SMCs lining the luminal surface and the pit region [14]. These dysplastic cells were almost entirely devoid of mucus as determined by periodic acid–Schiff (PAS) staining [14], which is characteristic of neutral mucins such as MUC5AC. Paradoxically, *Muc5ac* transcripts, but also *Muc6* transcripts, particularly in the antrum, were significantly increased in 20-week-old *Tff1^KO^* mice when compared with the wild type animals [123]. Furthermore, Tff2 expression was strongly reduced in the stomach of *Tff1^KO^* mice, particularly in the corpus [14,123,135]. All *Tff1^KO^* mice developed antral/pyloric adenoma, and in about 30% of the animals, carcinomas in situ were detected at 5 months of age [14].

A systematic analysis described the morphological changes in the antral mucosa including hyperplasia, dysplasia, localized neoplasia (carcinoma in situ), and invasive neoplasia [136]. In 60-day-old *Tff1^KO^* mice, the hypertrophic pits had already lost most of their PAS staining, but consisted mainly of proliferating, undifferentiated cells typical of progenitor cells [136]. By 6–12 months, dysplasia was evident, and carcinoma in situ was observed by 12–17 months together with signs of submucosal invasion [136]. These invasive cells mainly represented a mixed population of highly proliferative isthmus progenitor and stem cells, but also contained a few mature mucous and enteroendocrine cells [136]. Taken together, these results are an indication that the antral carcinoma of *Tff1^KO^* mice are of stem cell origin [136].

The age-dependent progression from hyperplasia to neoplasia in *Tff1^KO^* mice is accompanied by NF-κB-mediated inflammation and increased antral expression of *Cxcl1* and *Cxcl5* [123,137]. This is in line with the observed increase in leukocytes and monocytes/macrophages as well as an up-regulation of *Il-17* expression [138]. Of special note is that early treatment (3–12 weeks of age) of *Tff1^KO^* mice with the selective COX-2 inhibitor celecoxib reduced dysplastic lesions by 50% [137]. In contrast, treatment of older *Tff1^KO^* mice (between 1 month and 4 months of age) led to ulceration and infiltration of mononuclear inflammatory cells, specifically of the adenoma [139]. Genetic deletion of *Cox2* in *Tff1^KO^* mice reduced the adenoma size and ulceration with a chronic inflammatory reaction [140].

In contrast to wild type animals, *Tff1^KO^* mice were reported to show a predominant nuclear localization of β-catenin in the antropyloric region, which was accompanied by increased expression of the β-catenin/WNT target gene *c-Myc* [141]. Such a nuclear localization was also observed in MKN28 gastric cancer cells, and this nuclear localization was significantly reduced by treatment with a TFF1-conditioned medium [141]. This could be an indication that TFF1 acts extracellularly, as expected for a secretory peptide.

*N*-methyl-*N*-nitrosurea (MNU) is able to induce antral carcinogenesis in mice. This is associated with methylation and epigenetic silencing of *Tff1* [142]. Gastrin suppressed MNU-induced antral carcinogenesis by changing the epigenetic status of *Tff1*. Furthermore, heterozygous *Tff1*-deficient mice showed increased susceptibility to MNU-induced carcinogenesis [142]. Thus, the function of *Tff1* as a tumor suppressor clearly shows a gene dose effect.

### 4.3. Lineage Tracing Studies

Studies using a transgenic *Tff1-CreERT2* model (tamoxifen-inducible Cre recombinase) revealed rare tracing of SMCs in the fundic units, reflecting the distribution of endogenous Tff1 protein [143,144]. In contrast, about 20% of the antral units stained entirely positive, indicating that recombination also occurred in stem cells [143,144]. Thus, only in antral units is *Tff1* expressed also in stem cells, which repopulate the entire antral units during the process of self-renewal. This early expression in antral stem cells might also explain why *Tff1^KO^* mice develop adenoma and carcinoma exclusively in the antrum. Particularly promising candidates would be the Aqp5^+^/Axin2^+^/Lgr5^+^ population of stem cells at the gland base (Figure 1). Here, Tff1 peptide seems to play a role as a tumor suppressor.

### 4.4. TFF1 and Human Gastric Cancer

Remarkably, TFF1 expression is lost in about 40–60% of GCs and chromosome 21q22, i.e., the chromosome where all TFF genes are located in tandem, is commonly deleted [15,145,146]. Furthermore, somatic TFF1 mutations were described in GC [147,148]. However, systematic analyses revealed that promotor methylation and loss of heterozygosity, but not mutation, seem to underlie the loss of TFF1 in GCs [142,149,150].

### 4.5. Possible Molecular Function(s) of Tff1: Lectin-Triggered Receptor Activation/Blocking

Recently, four possible hypothetical models were proposed explaining the molecular function of TFF1 [13]. Moreover, a combination of these models is possible.

First, hypothetically, intracellular TFF1 could play a role for the correct folding, assembly, and secretion of the mucin MUC5AC in SMCs as both MUC5AC and TFF1 are co-secreted. This might explain why in *Tff1^KO^* mice, the unfolded protein response (UPR) is activated [151]. Interestingly, mice with missense mutations in the intestinal mucin *Muc2*, causing aberrant mucin assembly, also show ER stress and inflammation [152]. Furthermore, about 70% of *Muc5ac^KO^* mice spontaneously develop antropyloric hyperplasia and at least 17% develop adenomas but no carcinomas [153], which is reminiscent of *Tff1^KO^* mice [14]. Generally, TFF1 could function as an intracellular chaperone; also, the heterodimer with a yet unknown partner protein X (TFF1-X; M_r_ of X: 60k) might be an interesting candidate [122]. However, most of TFF1 clearly does not interact with mucins in vivo [122,123,124,154].

Second, monomeric TFF1 with the probably highly nucleophilic free thiol group at Cys^VII^ (Figure 4) was postulated to act as a scavenger for extracellular ROS/RNS, which might be particularly important for the protection of stem cells [12,122,123,124]. Here, the antrum might be more vulnerable as the number of proliferating cells is much higher and antral SMCs show a much higher turnover rate [6,25,26].

Third, a gastrokine 2 homodimer was detected specifically in *Tff1^KO^* mice, and here, particularly in the antrum, as the usual Tff1-Gkn2 heterodimer cannot by synthesized any more [123]. Hypothetically, this Gkn2 homodimer could account for dysregulated differentiation processes.

**Figure 5:**
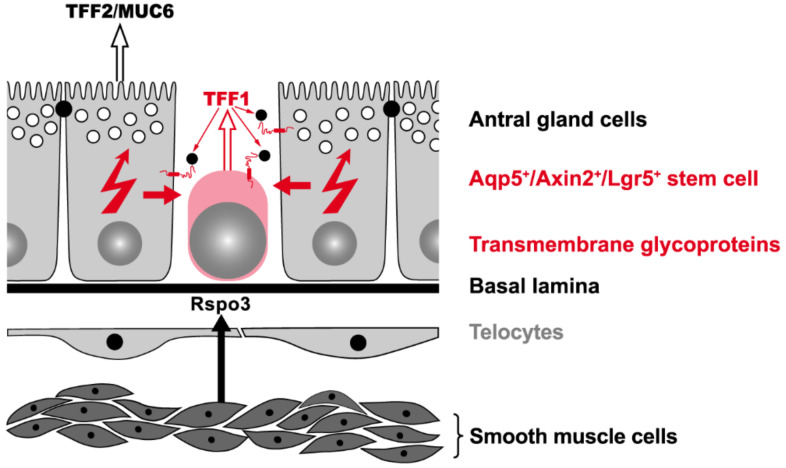
Schematic representation of an Aqp5^+^/Axin2^+^/Lgr5^+^ stem cell and its niche at the base of a murine antral unit and hypothetical model on the role of Tff1 for regulating antral gland differentiation and proliferation. Neighboring AGCs characteristically express A4gnt and secrete Tff2 and Muc6. Rspo3 from smooth muscle cells beneath the gland base induces secretion of Tff1 from the Lgr5^+^ stem cell. Depicted are also αGlcNAc glycosylated transmembrane proteins at the basolateral side of AGCs and on the Lgr5^+^ stem cell, respectively. Tff1 can bind as a lectin to these transmembrane glycoproteins triggering signaling events (Lectin-triggered Receptor Blocking Hypothesis [13]).

Fourth, TFF1 could bind as an extracellular lectin to the carbohydrate moiety of transmembrane glycoproteins. Of special note is that in such a lectin binding, TFF1 would act as a low affinity ligand, which is in agreement with the rather high concentration of TFF peptides (10^−6^–10^−7^ M or even higher) necessary to trigger weak motogenic and anti-apoptotic effects (details: [12]). Based on the in vitro binding studies [122,128], it is probable that the terminal αGlcNAc residue (recognized by the monoclonal antibody HIK1083 and the lectin GSA-II, respectively) is expected to be an essential part of the sugar binding epitope recognized by TFF1 [12,126]. For the biosynthesis of such a residue, the enzyme A4GNT is required (review: [93]). Of special note and in agreement with the in vitro binding studies, it has been documented in rats in vivo that intravenously injected ^125^I-TFF1 monomer (and also ^125^I-TFF2 and ^125^I-TFF3) binds specifically to A4GNT expressing cells, i.e., gastric MNCs, AGCs, and duodenal Brunner glands, which typically express MUC6 and TFF2 [155]. These studies using autoradiography of histological sections, imply that there are TFF1 binding sites at the basolateral side of MNCs and AGS, and there are even indications for a receptor-mediated transcytosis of TFF peptides to the apical mucus layer [156,157]. The basolateral TFF1 binding sites at MNCs and AGCs are probably glycosylated transmembrane proteins, which bear a terminal αGlcNAc residue at their carbohydrate moiety due to their characteristic A4GNT expression (Figure 5). From the autoradiographic studies [155], it cannot be excluded that Aqp5^+^/Axin2^+^/Lgr5^+^ stem cells in direct proximity to AGCs (Figure 5) also bind Tff1. Indeed, this assumption is supported by the observation that both mouse and human antral Aqp5^+^/Lgr5^+^ stem cells express A4gnt (and also Muc6 and Tff2) such as AGCs [41,46]. Thus, these basal antral Lgr5^+^ stem cells do not conform to the classical “undifferentiated” stem cell model.

From lineage tracing studies, it can be concluded that Tff1 is also expressed in antral stem cells [143,144]. This is in line with the observation that relatively high concentrations of Rspo3 from smooth muscle cells are able to induce differentiation of antral basal Lgr5^+^ cells towards a secretory cell type, which also releases Tff1 [46]. Tff1 could then communicate via its lectin activity with the neighboring AGCs in a paracrine manner. In a feedback loop, AGCs may respond to the stem cells in a manner that silences particularly the proliferative potential of Lgr5^+^ cells necessary to maintain homeostatic conditions. Notch signaling would be perfectly designed to regulate the delicate balance between the proliferation and differentiation of antral Lgr5^+^ stem cells. Indeed, Notch 1 and Notch 2 receptors are known to regulate antral homeostasis [61]. Moreover, components of Notch signaling pathways are enriched in Aqp5^+^/Lgr5^+^ stem cells [41]. From a mechanistic point of view, Tff1 might inhibit Notch, whereas Tff1-deficiency would then activate Notch, promoting proliferation.

Alternatively, autocrine stimulation of antral Lgr5^+^ stem cells by Tff1 also seems possible. Generally, AGCs are expected to play a role as a part of the niche for neighboring Lgr5^+^ stem cells (Figure 5) similar to fundic parietal cells [34] or small intestinal Paneth cells [57]. As a consequence, a loss of Tff1 would probably reduce, at least partially, the characteristics of AGCs as niche cells.

Based on the phenotype of *Tff1^KO^* mice, it is postulated that lectin binding of Tff1 is essential for proper differentiation of antral glands. Such a hypothetical mechanism might explain why the loss of extracellular Tff1 activates β-catenin signaling and promotes cell proliferation [141]. The observation that *A4gnt^KO^* mice show an even more severe phenotype than *Tff1^KO^* mice as all of the *A4gnt^KO^* mice develop antral adenocarcinoma, is completely in line with this lectin hypothesis [95].

Currently, the nature of the glycosylated membrane protein(s) mediating lectin-triggered Tff1 signaling has not been elucidated. There are a plethora of glycoproteins that are claimed to bind TFF peptides such as β1 integrin, CRP-ductin/DMBT1^gp340^, CXCR4, CXCR7, PAR2, PAR4, LINGO2, and LINGO3 (details and references: [12,13,158]). However, specific binding of TFF1 has not been convincingly documented yet. Generally, TFF1 could activate signaling of the postulated glycosylated transmembrane proteins or it could inhibit their signal transduction. Remarkably, there are repeated reports that TFF1, and also TFF2, block the activation of several receptors, such as the IL-1β receptor (by TFF2 [159]), Toll-like receptor driven suppression of IL-12 expression by TFF2 [160], tumor necrosis factor α receptor (TNFR1; by TFF1 [137]), TNFR2 (by TFF1 [161]), and IL6Rα-gp80 (by TFF1 [162]). The hypothetical mechanism, where signaling of receptors is inhibited by TFF peptides, was termed Lectin-triggered Receptor Blocking Hypothesis [13].

An elegant, and also quite simple, experimental set-up to test this hypothesis would by a trial to cure *Tff1^KO^* mice from developing adenoma and carcinoma by the oral application of a recombinant *Lactococcis* *lactis* strain secreting TFF1. Such a strain is commercially available (termed AG013) and has already been proven in a phase 1b study in patients with locally advanced head and neck cancer receiving chemotherapy [163]. *L. lactis* would be ideally suited to deliver recombinant TFF1 to the stomach as *Lactococcus* is oxygen tolerant belonging to the same order (*Lactobacillales*) as *Lactobacillus*, the latter being the predominant colonizer in the murine stomach [164].

## 5. Conclusions and Medical Perspectives

Taken together, self-renewal of fundic and antral units, respectively, is regulated by different stem cell populations. Reciprocal epithelial–mesenchymal interactions with underlying Foxl1^+^ telocytes of the lamina propria as well as smooth muscle cells of the lamina muscularis mucosae play an essential role. Probably, certain parietal cells in fundic units and AGCs in antral units have a function as niche cells for the neighboring stem cells. In the latter, the lectin TFF1 from Lgr5^+^ stem cells probably plays a role for their regulation in an autocrine or paracrine mode. Signaling is predicted to occur via a lectin interaction with a glycosylated transmembrane protein(s). This hypothetical model would explain why *Tff1^KO^* mice develop antral adenoma and carcinoma. Large progress was obtained within the last years in characterizing the different stem cell populations as well as the signals triggering their stepwise differentiation. Generally, there are multiple ways to gastric cancer, and carcinogenesis is a multistep process characteristically accompanied by inflammation as a key step (review: [70]). Here, already present gastric ILC2s, which are regulated in a sex-specific manner [22], as well as infiltrating macrophages, are of major importance. This explains, why anti-inflammatory therapeutic regimes are promising protection strategies as demonstrated in a number of mouse models (examples: [63,103,107,140,165]). In the future, much progress can be expected from the use of organoids as model systems [165]. A major goal will also be developing a better understanding of stem cell (dys)regulation in the setting of inflammation.

## Data Availability

Not applicable.

## Abbreviations

A4GNT	α1,4-*N*-acetylglucosaminyltransferase
AGC	Antral gland cell
AR	Amphiregulin
BMP	Bone morphogenetic protein
EGF	Epidermal growth factor
ER	Endoplasmic reticulum
FCGBP	IgG Fc binding protein
FOX	Forkhead box
GC	Gastric cancer
GKN	Gastrokine
IL	Interleukin
ILC2	Group 2 innate lymphoid cell
IM	Intestinal metaplasia
MNC	Mucous neck cell
PAS	Periodic acid–Schiff
PPI	Proton pump inhibitor
RNS	Reactive nitrogen species
ROS	Reactive oxygen species
SHH	Sonic hedgehog
SMC	Surface mucous cell
SPEM	Spasmolytic polypeptide expressing metaplasia
TFF	Trefoil factor family
TGF	Transforming growth factor
vENCC	Vagal enteric neural crest cell
